# Scimitar Syndrome: A Thorough Diagnosis in a Pediatric Patient

**DOI:** 10.7759/cureus.66302

**Published:** 2024-08-06

**Authors:** Eduardo Tomás Alvarado, Marisela Sarahi Zaragoza Martínez, Oscar Andres Ramirez Teran

**Affiliations:** 1 Pediatric Cardiology, Hospital General Regional 17, Cancun, MEX; 2 Pediatrics, Instituto Nacional de Pediatría, Mexico City, MEX

**Keywords:** dextrocardia, pediatrics, congenital heart disease, pulmonary hypoplasia, scimitar syndrome

## Abstract

Scimitar syndrome is characterized by the anomalous connection of the right pulmonary venous return to the hepatic portion of the inferior vena cava. Its name is derived from the characteristic image observed in chest X-ray, CT scan, or during pulmonary angiography in cardiac catheterization. It is more common among females and rarely affects the left lung. The importance of knowing its symptoms and presentation allows for a high diagnostic suspicion, thus avoiding the underdiagnosis of the disease. The prognosis is generally good, and timely diagnosis can prevent the occurrence of complications such as pulmonary hypertension or portal hypertension. We present the case of an eight-year-old female patient, who was previously evaluated for episodes of lower respiratory tract infections at 18 months of age, detecting only dextroposition, without any diagnostic workup. She was then sent to our office at eight years of age, with the onset of exercise-induced dyspnea. A comprehensive workup was conducted, with a diagnosis of scimitar syndrome.

## Introduction

Scimitar syndrome is a rare but well-characterized disease accounting for 0.5-1% of all congenital heart diseases. The term scimitar syndrome was coined by Neill et al. [1], with reference to the characteristic appearance of the anomalous right pulmonary venous confluence to the hepatic portion of the inferior vena cava, instead of draining into the left atrium. The image observed in chest X-ray or during pulmonary angiography in cardiac catheterization (due to the anomalous connection) resembles a curved Turkish sword called a scimitar. The X-ray appearance is referred to as the scimitar sign [2].

Hypoplasia of pulmonary arteries, pulmonary hypoplasia, pulmonary sequestration, as well as other cardiac malformations have also been described [2]. It is more common among females and rarely affects the left lung. Although its incidence is unknown due to a large number of asymptomatic patients, it is estimated to be between 1 and 3 per 100,000 live births [3]. In some cases, the abnormal venous drainage can be visualized on chest X-ray as a curved linear opacity parallel to the right heart border directed toward the cardio-phrenic angle [4].

## Case presentation

An eight-year-old female was brought for evaluation as she was having difficulty breathing during moderate physical activity in the last two weeks. At 18 months of age, dextrocardia was detected on a chest X-ray during the investigation of a lower respiratory tract infection. No further extension studies were performed, and no alterations were identified up to the time of her current symptoms.

There were no signs of cyanosis during the examination, and her weight and height were normal for her age. Heart sounds were rhythmic, no murmurs were auscultated, and the patient had adequate air entry and exit. There were no other added phenomena. There were no visceromegalies. Peripheral pulses were palpable, and there was no pedal edema, with a baseline saturation of 92%.

**Figure 1:**
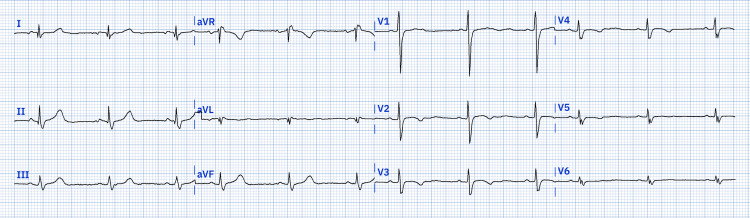
The 12-lead electrocardiogram. The 12-lead electrocardiogram shows a predominance of right cavities and an electrical axis of P with a deviation at -20 degrees.

Her electrocardiogram showed sinus rhythm in dextrocardia, P axis -20 degrees, aQRS -30 degrees, with a predominance of right forces, as shown in Figure 1.

**Figure 2:**
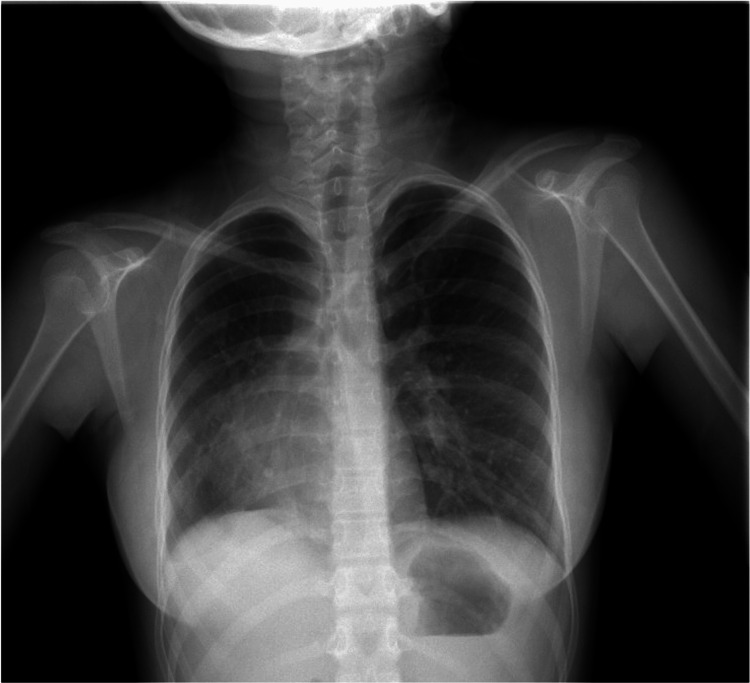
Chest X-ray posteroanterior view in scimitar syndrome. Chest X-ray taken at eight years of age shows dextroposition of the cardiac silhouette and a smaller right lung.

Her chest X-ray showed a reduction in the right lung parenchyma, dextrocardia, and increased parahilar vasculature, as shown in Figure 2.

A transthoracic echocardiogram revealed dextroposition, left apex, no septal defects, a partial anomalous connection of the infracardiac pulmonary veins to the inferior vena cava, peak gradient of 10 mmHg, adequate biventricular function, and no other structural abnormalities. Thus, the diagnosis of scimitar syndrome was reached.

**Figure 3:**
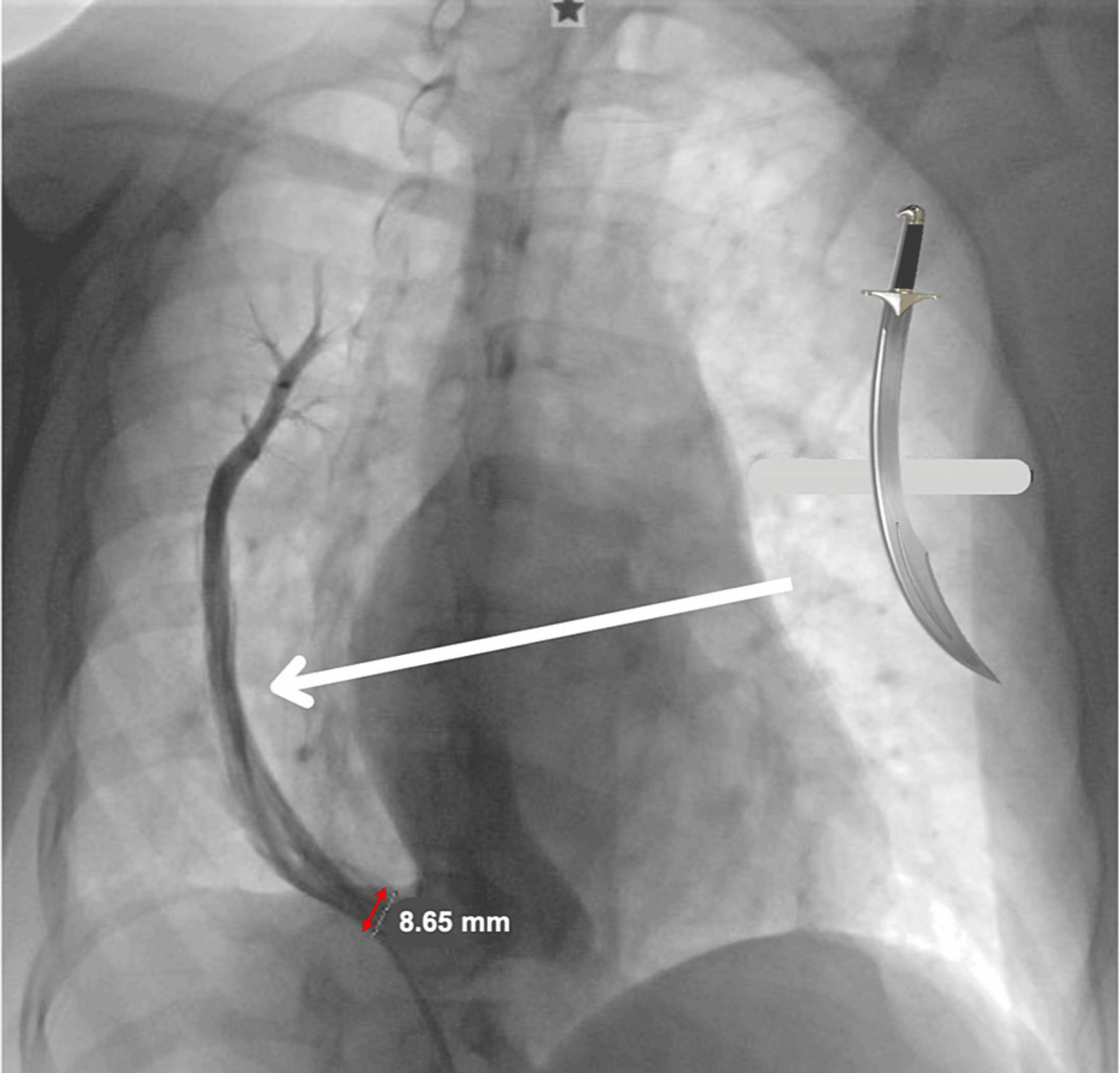
Characteristic image of scimitar syndrome on angiography. Angiography showing pulmonary sequestration of the right lower lobe and a partial anomalous connection of the right pulmonary veins (white arrow) to the inferior vena cava (double-headed red arrow). In the upper right part, its similarity to a scimitar (saber) can be observed.

To complete her workup, a diagnostic cardiac catheterization was performed, observing the characteristic image of scimitar syndrome on angiography, as shown in Figure 3.

Once the diagnosis was confirmed, it was decided to start treatment with an aldosterone receptor antagonist such as spironolactone at a dose of 1 mg/kg/dose every 12 hours. During the patient’s follow-up, in her consultations three and six months after starting the medication, she reported improvement in her symptoms, with a functional class I on the Ross scale. Her saturation also improved, maintaining records above 95% during the following visits. The patient was evaluated by the cardiothoracic surgery service, which determined that she did not require corrective surgical management at the time of the evaluation.

## Discussion

Scimitar syndrome represents 3-5% of cases of anomalous pulmonary venous return and occurs more frequently in males [5]. Female presentation, as is the case in our patient, has been reported less frequently.

Classically, two forms of scimitar syndrome have been described, an infantile form, mainly associated with vascular alterations or thoracic malformations, and the adult form, which is usually asymptomatic [6]. In the infantile presentation, the diagnosis is made due to the association with cardiac or major thoracic alterations. In our patient, findings such as dextroposition or the smaller size of the right lung in the chest X-ray could guide the diagnosis very early. However, during her evaluation at 18 months of age, only the alteration in the position of the heart was detected, without establishing any other type of approach or complementary study. The reason for the asymptomatic clinical course led to the suspicion and definitive diagnosis being made until the age of eight years, when the first symptoms appeared during physical activity.

The mean age of diagnosis reported by Brunet-Garcia et al. in their review of 10 cases diagnosed with scimitar syndrome was 10.4 months (range: 0.1-150.2) [7,8]. In the case we have presented, the diagnosis was made at the age of eight years. This highlights the importance of knowing and disseminating information on the topic, as diagnosis can be challenging without strong initial suspicion. Similar to five of the 10 patients reported by Brunet-Garcia et al., the diagnosis occurs incidentally or following repeated respiratory infections. It is important to mention that incidental radiographic findings are present in up to 41.2% [8] of the radiographs requested for another reason or different diagnostic suspicion, as in the case of our patient, as early as 18 months of age.

The presence of dyspnea or mild saturation decrease (92% in our patient’s case) is also key to the diagnosis. Mild desaturation may be related to the degree of pulmonary involvement or the number of anomalously connected pulmonary veins. In previous case reports, saturation was not mentioned as a suspicion or diagnostic criterion for scimitar syndrome [7,8]. Dextrocardia and apparent pulmonary hypoplasia should guide the workup to rule out associated congenital, vascular, or cardiac abnormalities.

Wang et al. reported that 43.5% of patients with scimitar syndrome were associated with another type of congenital heart disease such as atrial septal defect in 60-70% of cases and ventricular septal defect and coarctation of the aorta or tetralogy of Fallot in the other 25% [9]. Additionally, noting that pulmonary hypoplasia is more frequent when the diagnosis is made in pediatric age compared to adulthood (90% vs. 60%, respectively) [10].

Although it has been reported that the development of pulmonary hypertension occurs in 60-70% of patients with intracardiac defects, Robledo et al. presented the case of a 26-year-old female patient who developed pulmonary hypertension without the presence of previous symptoms [5]. Therefore, the appearance of symptoms during childhood could suggest the need for closer follow-up, given the possibility of developing complications in later stages, despite a clinical course with non-specific or mild data.

The prognosis is good in most cases. It can worsen if symptoms appear early, requiring evaluation for surgical treatment to reduce subsequent complications such as pulmonary hypertension or portal hypertension, always considering the risk of post-surgical stenosis. The appearance of mild symptoms during exercise and variations in saturation can guide clinical decisions allowing the onset of early pharmacological management. Family counseling is also an important part of follow-up.

## Conclusions

In pediatric patients with dyspnea and saturation below 95%, a chest X-ray is recommended. In the presence of dextrocardia and pulmonary hypoplasia, the presence of anomalous pulmonary vein connection must be ruled out. Physical examination, pediatric follow-up, chest X-ray, electrocardiogram, and cardiac CT are essential for diagnosing conditions and surgical planning in disorders such as scimitar syndrome.
